# Examining Facilitators and Barriers to Cardiac Rehabilitation Adherence in a Low-Resource Setting in Latin America from Multiple Perspectives

**DOI:** 10.3390/ijerph19041911

**Published:** 2022-02-09

**Authors:** Diana Marcela Rangel-Cubillos, Andrea Vanessa Vega-Silva, Yully Fernanda Corzo-Vargas, Maria Camila Molano-Tordecilla, Yesica Paola Peñuela-Arévalo, Karen Mayerly Lagos-Peña, Adriana Marcela Jácome-Hortúa, Carmen Juliana Villamizar-Jaimes, Sherry L. Grace, Hugo Celso Dutra de Souza, Adriana Angarita-Fonseca, Juan Carlos Sánchez-Delgado

**Affiliations:** 1 Universidad de Santander, Facultad de Ciencias Médicas y de la Salud, Bucaramanga 680003, Colombia; dianita-397@hotmail.com (D.M.R.-C.); andreevega@hotmail.com (A.V.V.-S.); yullycorzo@gmail.com (Y.F.C.-V.); camila7molano@gmail.com (M.C.M.-T.); yesikappa@hotmail.com (Y.P.P.-A.); mayerlylagos23@gmail.com (K.M.L.-P.); amjhortua@gmail.com (A.M.J.-H.); adriangarita@hotmail.com (A.A.-F.); 2 Rehabilitation Center, Research and Development Department, Bucaramanga 680003, Colombia; Carmenvillamizarj@hotmail.com; 3 Faculty of Health, York University, Toronto, ON M3J 1P3, Canada; sgrace@yorku.ca; 4 KITE-Toronto Rehab Institute, & Director of Cardiac Rehabilitation Research, Peter Munk Cardiac Centre, University Health Network, University of Toronto, Toronto, ON M4G 1R7, Canada; 5 Laboratory of Physiology and Cardiovascular Physioterapy, Ribeirão Preto Medical School, University of São Paulo, Ribeirão Preto 14049-900, Brazil; 6 Université du Québec en Abitibi-Témiscamingue, Rouyn Noranda, QC J9X 5E4, Canada; 7 Centre de Recherche du Centre Hospitalier de l’Université de Montréal, Montreal, QC H2X 0A9, Canada; 8 Grupo de Investigación Ser Cultura y Movimiento, Universidad Santo Tomás-Bucaramanga, Santander 680001, Colombia

**Keywords:** cardiac rehabilitation, secondary prevention, heart diseases, exercise, physical activity, health services accessibility, treatment adherence and compliance

## Abstract

Cardiac rehabilitation (CR) is under-used, particularly in low-resource settings. There are few studies of barriers and facilitators to CR adherence in these settings, particularly considering multiple perspectives. In this multiple-method study, a cross-sectional survey including the Cardiac Rehabilitation Barriers Scale (each item scored on a five-point Likert scale) was administered to patients treated between February and July, 2019, in three CR centers in Colombia. A random subsample of 50 participants was invited to a focus group, along with an accompanying relative. Physiotherapists from the programs were invited to an interview, with a similar interview guide. Audio-recordings were transcribed and analyzed using interpretive description. A total of 210 patients completed the survey, and 9 patients, together with 3 of their relatives and 3 physiotherapists, were interviewed. The greatest barriers identified were costs (mean = 2.8 ± 1.6), distance (2.6 ± 1.6) and transportation (2.5 ± 1.6); the logistical subscale was highest. Six themes were identified, pertaining to well-being, life roles, weather, financial factors, healthcare professionals and health system factors. The main facilitators were encouragement from physiotherapists, relatives and other patients. The development of hybrid programs where patients transition from supervised to unsupervised sessions when appropriate should be considered, if health insurers were to reimburse them. Programs should consider the implications regarding policies of family inclusion.

## 1. Introduction

Cardiovascular diseases (CVDs) are highly prevalent, and CVD patients have an increased risk of mortality and morbidity, such as rehospitalization, revascularization and other major adverse cardiac events [1,2]. The burden is particularly high in low- and middle-income countries; in Colombia, for example, CVD continues to be the leading cause of death [3], causing 26% of total deaths [3]. It is the second leading cause of death and disability (DALYs) combined in Colombia [3,4]. The annual healthcare cost for a person with CVD is between I$ 4277 and I$ 6616 [5].

Cardiac rehabilitation (CR) is a comprehensive outpatient program of exercise and education, designed to improve lifestyle, control risk factors and implement secondary prevention recommendations [6]. Participation in these programs is associated with an approximately 20% lower morbidity and cardiovascular mortality [7], and reduced healthcare costs.

Despite these benefits, less than 30% of the eligible population has access to CR; of these, approximately 50% drop out [8,9]. Barriers to CR use have been well characterized in the Western literature, including factors at the provider and patient levels [10]. There are, however, relatively few studies of these barriers in lower-resource settings, where barriers are likely even greater [11,12]. Indeed, a 2017 review identified only 13 studies assessing CR barriers in low- and middle-income countries; of these, 7 were in Latin America, with 1 in Colombia published over 10 years ago [12,13]. There have been some other more recent studies in low-resource settings using the psychometrically validated Cardiac Rehabilitation Barriers Scale (CRBS) [14,15,16,17,18,19,20]. Many of these studies have been solely qualitative and/or have not considered the perspectives of multiple stakeholders. For instance, in addition to considering barriers at the patient and provider levels, it would be important to consider the issues faced by informal caregivers who are supporting patients to come to sessions and fully engage in them. In addition, many of these studies have not considered facilitators which, if identified, could be leveraged to increase participation. Therefore, the purpose of this study was to explore barriers and facilitators to CR adherence from the perspectives of patients, their informal caregivers and CR providers, in a Latin American city.

## 2. Materials and Methods

### 2.1. Design and Procedure

This study used multiple methods, with an explanatory sequential design [21,22,23]. The quantitative component involved a cross-sectional survey of patients in three CR centers in Bucaramanga, Colombia.

The subsequent qualitative portion comprised a focus group (patients) and interviews (physiotherapists and relatives), conducted in Spanish. One investigator led the focus group, while another took notes. Focus groups and interviews were audio-recorded.

### 2.2. Setting

The three CR centers where recruitment took place were coordinated by a physiotherapist. There were three physiotherapists working at these centers. The programs were accessible to patients by public and private transportation.

CR sessions are held 5 days a week, consisting of 4 phases: warm up, structured aerobic exercise on a treadmill or bike, balance and stretching exercises, as well as a cool down. It is mandatory for patients to attend each session with a relative. Additionally, the programs offer educational sessions delivered by a multidisciplinary team. The number of sessions prescribed to each patient ranges between 15 and 30.

### 2.3. Participants

For the quantitative portion of the study, CR patients from the three centers treated between February and July 2019 were invited to participate. For the qualitative portion of the study, a random subsample of 50 participants from two of the centers who completed the survey were invited to participate in the focus group. Relatives of these 50 were all invited to the focus group. Physiotherapists, the most common CR providers in the country, from the two programs were also invited for interview [24].

The patient eligibility criteria were a diagnosis of CVD, 18 years of age or older, reside in Bucaramanga, and were native Spanish speakers. Inclusion criteria for relatives were being over 18 years old, being within a maximum of three degrees of relation to the CR participant, living with or taking care of the patient, and accompanying the patient to at least three CR sessions. Inclusion criteria for physiotherapists were being a CR specialist at one of the two centers, and more than six months’ experience in CR. Participation in the study was voluntary, and all participants provided signed informed consent.

### 2.4. Instruments

For the quantitative part of the study, sociodemographic and clinical characteristics were assessed and the CRBS [20] was administered. This was via self-report or through an interview where patients were unable to complete the survey, in which case, the administrator maintained a neutral stance.

The CRBS is a psychometrically validated tool assessing how patient, provider and health system-level factors impact CR participation [20]. The scale has been translated and adapted to Spanish in Colombia, with acceptable psychometric properties (Cronbach’s α = 0.84; Intra-class correlation = 0.71) [18,19]. The CRBS consists of 21 items in 4 subscales: perceived need for CR/healthcare factors, logistic factors, work/time conflicts, and comorbidities/functional status. Items are scored on a 5-point Likert-type scale, from 1 “strongly disagree”, to 5 “strongly agree”; higher scores indicate greater barriers.

For the qualitative sub-study, a semi-structured guide was created for the focus group (which was subsequently used for the interviews), to explore barriers as well as facilitators to attending CR in depth. Six main categories and subcategories were explored: physical and psychosocial well-being, life roles (occupation/work, responsibilities at home), environmental factors (weather), financial factors (transportation cost, other financial responsibilities), healthcare provider factors (cardiologist/internist/sports doctor), and health system factors (location of the rehabilitation site, group exercise, insurance). In addition to the patients, relatives and therapists were also asked their sociodemographic characteristics.

### 2.5. Data Analysis

Quantitative analysis was performed in Stata 12.0 (Stata Corp LCC, College Station, TX, USA); descriptive examinations were performed.

For the qualitative analysis, focus group and interview audio-recordings were transcribed and coded, based on the main categories outlined above, using interpretive descriptive analysis [25]. Illustrative quotes were translated to English.

## 3. Results

### 3.1. Respondent Characteristics

Table 1 shows the participant characteristics from the quantitative (*n* = 210) and qualitative (*n* = 9) sub-studies. Forty-six (22%) participants completed the survey via self-report.

From the 20 (40%) people that confirmed attendance for the focus group, only 4 attended (20%; 3 patients and 1 relative). For that reason, after the focus group, semi-structured family interviews were undertaken (also led by the investigator), on the phone (also audio-recorded), using the same interview guide, with willing no-shows until saturation was achieved. Through this, six further patients and two relatives consented. Three physiotherapists also consented to be interviewed (*N* = 15 participants total).

The 3 relatives interviewed were wives of patients, with a median age of 54 (range = 50–60) years. The physiotherapists had a median age of 38 (range = 37–41) years, with 6, 12 and 14 years of experience in CR.

### 3.2. Quantitative Results

Table 2 shows CRBS items and subscales scores. Note that using Wilcoxon tests, total CR barriers score was unrelated to age and referral indication. There was a trend towards higher scores in women than men (*p* = 0.07), and there were significantly higher scores in those of lower socioeconomic status (*p* = 0.001).

As shown, items with the highest scores were “Cost”, “Distance” and “Transportation”. Logistical factors were the greatest barriers. The items with the lowest scores, suggesting they are hindering attendance least, were preferring to manage their CVD independently, as well as some of the items around the logistics of being referred, not perceiving they need it and older age.

### 3.3. Qualitative Results

Main barriers and facilitators, by respondent, are shown in Table 3. Some key quotes supporting each of these main factors are outlined below.

#### 3.3.1. Physical and Psychosocial Well-Being

Most of the participants agreed that comorbidities were not an impediment to attending CR. “I have back pain, so I have to stop and stretch. But no matter what, I arrived to my therapy sessions. That comes within each person, you have to prioritize CR and attend” (Patient #2).

Regarding psychosocial aspects, the importance of the accompanying relative as a facilitating agent for participation in the CR programs was highlighted. Patient #2 said: “When the family motivates you, it is easier to attend the program”. Physiotherapists noted the benefits and drawbacks, saying, for example: “Yes, especially for patients who live alone, men who are quite depressed and normally are alone, there is no one to accompany them” (Therapist #1).

#### 3.3.2. Life Roles

Among participants, occupation/work was not perceived as a barrier to attending CR. For example, they stated: “...I am not currently working. I am retired” (Patient #8). However, physiotherapists indicated that occupation/work can be a barrier when the patient is not retired and is not on sick leave: “There are many patients who are working, who start at 7 am and leave at 6 in the afternoon. If cardiac rehabilitation doesn’t start during sick leave, it is difficult to engage them…” (Therapist #3).

Regarding home duties, Patient #3 suggested that they are not barriers: “Yes, so, I’m in charge of my mother, but we are a large family. So they know that when I leave for therapy, they are going to take care of my Mom”. Likewise, Relative #3 stated: “It is not just the two of us, and I take care of him and I am the one who accompanies him everywhere”. On the other hand, Therapist #1 indicated that it could be a barrier: “Especially the grannies, I have heard that they have to take care of their grandsons...” (Therapist #1).

#### 3.3.3. Environmental Factors

Patients and family did not perceive the weather as a barrier. “I used to come at 11 in the morning. At that time it sometimes drizzled, but it didn’t matter” (Patient #2). “No, there is no problem, because he wasn’t worried about coming to his therapy... even if it rained” (Relative #1). In contrast the above, Therapist #2 indicated: “Yes, when it’s raining heavily they do not show up around here. Heat is not a problem, but water is...”.

#### 3.3.4. Financial Factors

Financial factors were a strong barrier to attending, especially transportation costs, because patients had to be accompanied. As Patient #1 stated: “some days I would get a taxi, and then I would come by bus, but it was always money”. Relative #2 exhorted: “The doctor always tells you that if you come alone, they won’t treat you”. Therapist #2 declared: “Patients who come from afar have to take a taxi, then that is an important barrier…Many people are poor and from rural areas; Of course it limits participation.”

#### 3.3.5. Factors Related to Health Professionals

Health professionals, such as cardiologists and internists, are perceived by physiotherapists and patients as facilitators to CR engagement. They assert the importance of CR and refer patients to it. “Yes, he explained the program to me and referred me. So after that, I started rehabilitation” (Patient #4). “Here, patients arrive on their own, but they arrive with a physician’s order” (Therapist #2). Likewise, they recognized the importance of other professionals in promoting CR attendance such as nutritionists, nurses and psychologists.

Finally, patients and relatives reported that physiotherapists are a facilitator, because they guide the rehabilitation process, and encourage patients to attend. “The therapist was very respectful. She was always keeping an eye on me, taking care of everything… my exercises, everything” (Patient #5). As Relative #1 stated: “Of course, physiotherapists are so important … he tells me that he felt better because of them.”

#### 3.3.6. Health System Factors

Group exercise was identified as the main facilitator. Carrying out team activities allowed patients to share, interact and meet other people experiencing the same situation. As Patient # 5 stated: “I really like friends, making friends”. Relative # 1 avowed: “In a group it seems to me that he gets more excited. Most of the sessions have been done in group and he has been motivated”. Therapist # 2 expressed: “It is important that it’s done in group, because they identify that they are not the only ones who are sick... A support for them emotionally. Group work is best; it doesn’t make sense to do it alone”.

Regarding the location of the CR clinics, it was perceived as a barrier by most of the participants. As Patient #7 stated: “Yes, you have to take a taxi or come in your own car because there is no bus”. As Relative #3 expressed: “It is not such a limitation I stop coming, but it is difficult because I live in another city. It would be better if the services were closer” (Relative #3). Finally, as Therapist #2 said: “For some, yes, for others it is a little difficult, although it has already improved with the bus route... There is still a way to improve in terms of mobility and accessibility.”

Participants indicated that one of the most important barriers is health insurance. The authorization process is long, and they usually do not approve the full number of sessions prescribed by the physician. As Patient #6 stated: “I stood in line. I waited about two hours for them to give me the authorization”. Relative #3 announced: “There is a long waiting list for appointments. Also they do not answer the telephones. You have to insist and insist”. This was reinforced by Therapist #2 who stated: “All the paperwork they have to do… then every 5 sessions they have to return and do all the authorization process once again”.

### 3.4. Integration

**Figure 1:**
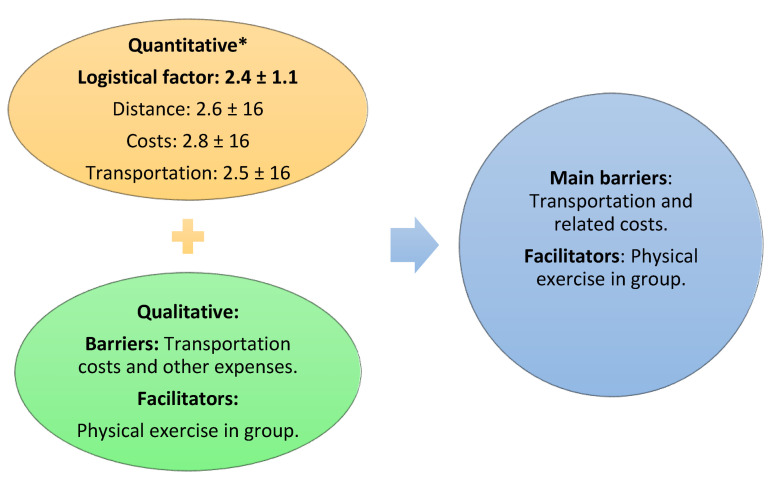
Triangulation of quantitative and qualitative results. * scored on a scale from 1 to 5, with higher scores indicating greater barriers [18].

By triangulating quantitative and qualitative results (Figure 1), it was concluded that costs, mainly due to the cost of transportation, were the greatest barriers. The facilitating factor that could increase participation and adherence to CR was “physical exercise in a group”.

## 4. Discussion

The results of this rare multiple-method study of multi-level CR barriers and facilitators in a low-resource setting show that logistical factors, such as cost, distance and transportation, are those which most affect adherence in CR. Healthcare professionals and engagement with other CR participants were motivating factors. Indeed, other studies have found that physiotherapists are central to supporting patients in their secondary prevention journey, especially when they experience kinesophobia [26,27,28].

The most prevalent perceived barriers identified in this study were generally consistent with other studies. However, there were some differences; as in some other studies, the most prevalent barriers were related to functional status/comorbidities (which surprisingly were downplayed by this cohort) and perceived need [18,29,30]. This is likely due to the proportion of enrollees in the samples, which also explains why the mean barrier scores were moderate to low. Costs were likely at the forefront given the low-resource setting, although direct and indirect costs associated with CR participation have been identified as barriers in many other studies [17,31]. Indirect financial barriers were of particular importance in this study, considering that almost 40% of the sample was unemployed or did not have any income. Taking a taxi to sessions, and paying for two persons on public transit each way, certainly seems prohibitive for patients of low socioeconomic means, who are also trying to pay for cardiac medications.

A unique finding of this study related to the role of having a family member accompany the patient to CR. Although this is an option in many programs, it is not generally mandatory [24]. Requiring a relative to attend may raise issues around privacy and confidentiality, and could take up space for additional patients in smaller centers, and as identified herein, may raise barriers in terms of the availability of a relative to accompany the patients (they may need to work inside or outside the home), and additional transportation costs; on the other hand, engaging supportive informal caregivers will likely promote the application of CR principles in the home on non-CR days, and also the maintenance of CR-related heart health behavior changes post-program. Moreover, given what is known about the impact of environments on health behavior, it is likely that informal caregivers were engaging in the same CVD risk behaviors which lead patients to develop heart disease; thus, by intervening with these family members, their health will also likely benefit [32]. Programs may wish to consider their policies regarding family inclusion, and where possible, make it flexible but not mandatory, so patients can make a decision that is best for their situation.

Another unique aspect of the study, in addition to the use of multiple methods to investigate the issue of CR adherence in this low-resource setting, was the capture of multiple perspectives. The family members’ comments generally reinforced those of the patients, as did those of the physiotherapists. However, in some instances, the physiotherapists added their knowledge about some of the other barriers experienced by patients who are not as adherent to CR.

### Limitations and Directions for Future Research

The primary limitation relates to generalizability (for the quantitative data; generalizability is not a goal of qualitative research). The sample was obtained from a single region of the country and only in three centers; therefore, the results may not be generalizable to all of Colombia, Latin America or low-resource settings more broadly. Moreover, the response rate was low. There may have been selection bias because those interested in the survey may be different from the average patient. It is possible the most motivated patients may have participated, or those who find it most difficult to participate in CR. The sample primarily comprised male urbanites; therefore, barriers and facilitators in women and those living in rural areas require more study. The study did not consider patients eligible for CR but who were not participating in a program. Therefore, our results are generalizable to patients attending CR who had not dropped out. Future research is needed to more fully understand barriers and facilitators for those not accessing CR or dropping out.

Another limitation relates to the mode of administration of the CRBS. Many patients provided their responses interview-style rather than self-report; this may have resulted in socially desirable responding or the under-reporting of barriers. However, given the consistency of results with other samples, this is not a major concern.

Caution is warranted in interpreting the results, because it was difficult to convene patients for a focus group; thus, the purposive sample was smaller than desired. However, through the subsequent interviews, it was possible to confirm and refine themes, and ultimately, theme saturation was attained. Themes were not generated by the type of participant, however; therefore, it should not be concluded that the themes represent all issues of importance to providers or relatives. Finally, due to the nature of the study design, it is not possible to establish causality.

Despite the above limitations, there are several important implications of this work. First, the administrative burden on patients for health insurance coverage was high. Working with insurance companies to streamline or digitize processes would certainly benefit patients. Rain was also a deterrent to CR participation. Physiotherapists could potentially develop home exercise prescriptions for patients on rainy days that do not require much space or equipment (e.g., aerobics, therabands, yoga). Given the centrality of support in promoting program adherence, results do not suggest that fully home-based programs would be a solution for these patients who have been able to access a program, although a hybrid program may be a good solution (i.e., some sessions on-site and others at home, potentially supported through technology) [33]. However, it would be important that insurance companies covered these services, and more evidence around the best mix of home and supervised programming, by element, is needed.

## 5. Conclusions

In low-resource settings, patients that do adhere to CR often do so due to support from healthcare providers, other patients and relatives. Logistical barriers nevertheless remain paramount, chiefly related to transportation and costs. This suggests that hybrid programs whereby patients can come on-site fewer days per week may optimize adherence, as long as healthcare insurance would cover this without any great administrative burden on patients. Programs should carefully consider policies around the inclusion of informal caregivers.

## Figures and Tables

**Table 1:** Sociodemographic and clinical characteristics of the patient sample, by sub-study.

Variable		Quantitative		Qualitative	
		*n* = 210	%	*n* = 9	%
Sex	Male	140	67	6	67
Age	Median (SD)	65	12	63	33
Residence	Rural	19	9	1	11
	Urban	187	91	8	89
Marital Status	Single	30	14	0	0
	Divorced	23	11	2	22
	Married	92	44	6	67
	Widow/er	39	19	0	0
	Common-law	22	11	1	11
	NR	3	1	0	0
Socioeconomic Status	Low	145	69	7	78
	Medium, High	61	29	2	22
	NR	3	1	0	0
Health Insurance *	Subsidized	36	17	3	33
	Contributive	154	74	4	45
	Special/Pre-paid	18	9	2	22
	NR	1	<1	0	0
Work Status	Employee	63	30	2	22
	Study and work	34	16	0	0
	Home-maker / unpaid	62	30	2	22
	Retired	14	7	0	0
	On disability or retired	17	8	5	56
	Unemployed	19	9	0	0
Education Level	None/Primary school	87	42	3	33
	Middle school	48	22	3	33
	Technician	16	8	2	22
	Post-Graduate	58	28	1	11
Household Living Arrangement	Alone	10	5	0	0
	Husband/wife	83	40	6	67
	Sons	41	20	2	22
	Other Relatives	70	33	1	11
	Friends	5	2	0	0
CR Indication	AMI/ACS	103	49	5	56
	Bypass	23	11	2	22
	Angioplasty	44	21	0	0
	Valvopathy	17	8	2	22
	Syncope	6	3	0	0
	Other §	16	8	0	0
Physical Disability	No	196	94	9	100
	Yes	13	6	0	0
Number of CR Sessions Attended	1–11	141	67	3	33
	12–23	37	18	1	11
	24–36	19	9	1	11
	More	7	3	2	22
	NR	5	2	2	22

AMI: acute myocardial infarction; ACS: acute coronary syndrome; CR: cardiac rehabilitation; SD: standard deviation; NR: not reported. * Contributory: funded using 12.5% of an individual’s salary; subsidized: insurance for those who cannot afford to pay into the contributory scheme is funded by the federal government; pre-paid medicine: privately funded, voluntary health insurance which offers additional coverage over-and-above the basic plan; special: for public teachers, the armed forces and workers of the state oil company; § includes heart failure, stable angina, and patients with pacemakers.

**Table 2:** CRBS item and subscale scores.

Barrier	Mean	SD	Median	IQR
Distance	2.6	1.6	2	1–4
Costs	2.8	1.6	3	1–4
Transport	2.5	1.6	2	1–4
Family responsibilities	1.9	1.2	1	1–2
I didn’t know what CR was	2.3	1.5	2	1–4
I don’t need CR	1.6	0.9	1	1–2
I already exercise at home, or in my community	2.1	1.3	2	1–3
Climate conditions	2.1	1.4	1	1–3
I find exercise tiring and/or painful	1.8	1.2	1	1–2
Lack of time	1.9	1.2	1	1–2
Of work responsibilities	1.7	1.2	1	1–2
Lack of energy	1.9	1.2	1	1–2
Other health problems	1.9	1.3	1	1–3
I feel old	1.6	1	1	1–2
My doctor didn’t refer me	1.6	1	1	1–2
A lot of people have heart problems and don’t attend	1.4	0.7	1	1–2
I can manage my problem and don’t need help	1.3	0.6	1	1–2
I think I was referred but they didn’t call me	1.4	0.8	1	1–2
A lot of time passed before I could get in the program	1.7	1.2	1	1–2
I prefer to take care of my own health, not in group Subscales	1.6	1	1	1–2
Perceived need/ healthcare factors	1.7	0.6	1.6	1.1–2.1
Logistical factors	2.4	1.1	2.2	1.4–3.2
Work/time conflicts	1.8	1.0	1.5	1–2
Comorbidities/ functional status	1.8	0.8	1.8	1–2.3
Total	1.9	0.6	1.9	1.4–2.3

CRBS: Cardiac Rehabilitation Barriers scale; SD: standard deviation; IQR: interquartile range.

**Table 3:** Most prevalent barriers and facilitators from qualitative interviews, by source.

Barriers	Facilitators
Weather ^#^	Physiological factors ^+,^*
Transportation cost ^+,^*^,#^	Cardiologist and internist physicians ^+,^*
Other costs ^+,^*	Physiotherapist ^+,#^
Health insurance ^+,^*^,#^	Group exercise ^+,^*^,#^
Clinic location *^,+^	

^+^ Patient; * Physiotherapist; ^#^ Relative.

## Data Availability

Data are available upon request due to privacy/ethical restrictions.
